# Fluid and electrolyte balance following consumption of skimmed milk and a plant-based soya beverage at rest in euhydrated males

**DOI:** 10.1007/s00421-024-05516-0

**Published:** 2024-05-29

**Authors:** Donald L. Peden, Seamus Derbyshire, Mark P. Funnell, Chris J. McLeod, Penny Rumbold, Emily Hansell, Tom Clifford, Stephen A. Mears, Lewis J. James

**Affiliations:** 1 National Centre for Sport and Exercise Medicine, School of Sport, Exercise and Health Sciences, Loughborough University, Loughborough, UK; 2 NIHR Applied Research Collaboration East Midlands, Diabetes Research Centre, University of Leicester, Leicester, UK; 3 Department of Sport, Exercise and Rehabilitation, Faculty of Health and Life Sciences, Northumbria University, Newcastle upon Tyne, UK

**Keywords:** Beverage hydration index, Hydration, Dehydration, Plant-based, Vegan, Vegetarian

## Abstract

**Purpose:**

Cow’s milk is one of the most hydrating beverages, but many individuals choose not to consume dairy in their diet due to intolerance, allergy, or dietary preference. Milk is commonly replaced with plant-based beverages, including soya which has the most comparable protein content, but little is known about their hydration potential. This study compared fluid and electrolyte balance responses between a soya beverage and skimmed cow’s milk.

**Methods:**

Ten healthy males [age 27 (6) y; body mass index 24.6 (2.3) kg/m^2^] completed two randomised counterbalanced trials, involving consuming 1000 mL water from approximately isocaloric amounts of skimmed cow’s milk (MILK) or a sweetened soya beverage (SOYA), in four aliquots over 30 min in a euhydrated fasted state. Volume, specific gravity, and electrolyte (sodium, potassium, chloride) concentrations were determined in total-void urine samples collected pre-/post-beverage ingestion, and hourly for 180 min thereafter. Hunger, thirst, nausea and stomach fullness were rated proximal to urine samples.

**Results:**

Total urine mass (MILK, 986 ± 254 g; SOYA, 950 ± 248 g; *P* = 0.435) and urine specific gravity (*P* = 0.156) did not differ between trials. Potassium balance was greater in SOYA 0–180 min post-beverage (*P* ≤ 0.013), whilst chloride balance was greater in MILK 0–120 min post-beverage (*P* ≤ 0.036). Sodium balance (*P* = 0.258), total electrolyte balance (*P* = 0.258), and subjective measures (*P* ≥ 0.139) were not different between trials.

**Conclusion:**

Replacing cow’s milk with a soya beverage did not negatively impact fluid balance in healthy young males, making it a viable option for those who choose not to consume dairy in their diet.

## Introduction

Water is the largest component of the human body, accounting for approximately 40–70% of an adult’s body mass. Despite this abundance, body water is tightly regulated, with counter-regulatory processes initiated following a change of as little as 1–2% body mass (Knepper et al. 2015). Adequate daily water intake recommendations vary globally, but are typically in the range of 2.5–3.7 L/day for adult males and 2.0–2.7 L/day for adult females (IOM 2005; EFSA 2010), although many adults fail to meet these guidelines (Kavouras 2019; Perrier 2017). Hypohydration represents a reduction in total body water, and whilst substantial hypohydration is rare, mild hypohydration or underhydration (Kavouras 2019) can more easily occur due to inadequate fluid intake, sweating or diuresis. Indeed, hypohydration may impact health outcomes (Carroll and James 2019; Clark et al. 2016; El-Sharkawy et al. 2015; Hooton et al. 2018; Jacques et al. 2021; Liska et al. 2019), as well as impair physical (James et al. 2019; Minshull and James 2013) and cognitive (Wittbrodt and Millard-Stafford 2018) performance, making the maintenance of day-to-day hydration vital.

As water intake is often below current recommendations (Kavouras 2019; Perrier 2017), the amount of a beverage retained in the body, rather than excreted in urine, could be an important factor for maintaining optimal hydration. In 2016, Maughan and colleagues developed the beverage hydration index (BHI), providing information about the amount of a given beverage that contributes to the maintenance of body water. Maughan et al. (2016) found that only a specifically designed oral rehydration solution and cow’s milk (both skimmed and whole options) had a significantly greater BHI than still bottled water (i.e., increased water balance vs still water). Cow’s milk is a complex beverage containing appreciable amounts of sodium, carbohydrate and protein (James et al. 2019). However, the milk protein content likely explains much of the positive effect of milk on hydration, since milk protein (James et al. 2011, 2013), but not whey protein (James et al. 2012, 2014; Hobson and James 2015) increases fluid balance, at least after exercise-induced dehydration.

Cow’s milk is commonly consumed by adults and children (Zhang et al. 2021; Green et al. 2015), making an important contribution to daily water intake, but its consumption is not possible for all. Globally, only 35% of the adult population has lactase persistence (the persistence of intestinal lactase until adulthood) needed for digestion of lactose (del Carmen Toca et al. 2022). Furthermore, veganism (an exclusively plant-based diet) is a growing dietary/lifestyle choice, with increasing sales of vegan products across the UK and North America (Sexton et al. 2022). For these reasons, as well as those related to personal and planetary health (Craig et al. 2023), plant-based dairy-milk alternatives have become common, including derivatives of soya, rice, oat, and various nuts. Soya-based beverages are the among the most popular and well-researched plant-based milk-alternatives (Hidalgo-Fuentes et al. 2024), having been used as a milk-alternative for at least 2000 years (Sethi et al. 2016). Additionally, and uniquely among plant-based milk-alternatives, soya beverages have comparable protein content to cow’s milk (James et al. 2019). However, like whey, soya protein is a fast-digesting protein (Tang et al. 2009), and so may act more like whey protein from a hydration perspective, but to date, the effect of plant-base milk-alternatives on hydration outcomes, including soya-based beverages, remains unclear.

Therefore, the aim of this study was to compare fluid and electrolyte balance responses following the consumption of skimmed cow’s milk and a sweetened soya beverage at rest. It was hypothesised that skimmed milk would produce better fluid balance (i.e. reduced urine output) than the soya beverage due to its milk protein content.

## Methods

### Participants

After approval by the Loughborough University Ethics Approvals (Human Participants) Sub-Committee (LEON-2021-6238), 10 healthy males [mean (standard deviation)] age, 27 (6) y; body mass, 78.5 (8.6) kg; stature 1.79 (0.04) m, body mass index 24.6 (2.3) kg/m^2^ completed the study. One additional male participant started the study but was withdrawn due to having diarrhoea after drinking the cow’s milk. Females were eligible to participate, but none were recruited into the study before the final sample was collected. An *a-priori* sample size calculation using GPower 3.1 was made using an α of 0.05, a β of 0.2 (statistical power of 0.8), the data from Maughan et al. (2016) for expected urine output (primary outcome) and a correlation between repeated urine output responses of 0.69 from rehydration studies from our laboratory. This sample size calculation determined 10 participants would be required to detect a 20% difference in urine output between the trials.

### Study overview

Participants completed an initial screening visit to provide written informed consent, complete a medical screening questionnaire, and recorded body mass (Adam Equipment Co., AFW-120K, Milton Keynes, UK) and stature (Seca Stadiometer, Birmingham, UK). Participants then completed two trials in which they consumed a volume of skimmed cow’s milk (MILK) or sweetened soya beverage (SOYA) providing 1000 mL water over 30 min, with all urine collected for the following 3 h. Trials were conducted in a randomised counterbalanced order and separated by ≥ 7 days.

### Pre-trial standardisation

In the 24 h before the first experimental trial, participants recorded dietary intake and any light physical activity, repeating these patterns in the 24 h preceding the second experimental trial. Additionally, participants were asked to refrain from moderate-to-vigorous intensity exercise and consuming alcohol in the 24 h pre-trial. Experimental trials commenced in the morning (time standardised within participant) following an overnight fast of ≥ 10 h, with 500 mL water ingested 2 h before arrival.

### Experimental trials

Upon arrival, participants confirmed compliance with pre-trial standardisations and provided ratings of hunger, thirst, nausea and stomach fullness using a 0–10 Likert scale (0 anchored with ‘not at all’ and 10 anchored with ‘extremely’), before they provided a total void urine sample (-30 min). Participants then consumed either MILK or SOYA over a 30 min period, in four equal aliquots consumed at 0, 7.5, 15 and 22.5 min of the 30 min period, with each aliquot consumed within 7.5 min. At the end of this 30 min period (0 min), and hourly thereafter for the next 3 h (60 min, 120 min and 180 min), participants provided subjective ratings and provided a total void urine sample. If participants needed to void their bladder during the hour, this urine sample was collected into the same collection vessel as the urine sample provided at the end of that hour. Additionally, at 0 min, participants rated the beverages for how ‘pleasant’, ‘thirst quenching’ and ‘refreshing’ they found them on 0–10 Likert scales (0 anchored with ‘not at all’ and 10 anchored with ‘extremely’). Laboratory temperature and relative humidity were recorded at − 30, 0, 60, 120 and 180 min.

### Experimental beverages

Beverages (Table 1) were skimmed cow’s milk (MILK; Sainsbury’s skimmed milk, J Sainsbury PLC, London, UK) and a plant-based soya beverage (SOYA; Alpro Soya, Alpro Group PLC, Birmingham, UK). Before the study, the density of the beverages were determined in three samples of each from different, newly opened bottles by weighing the mass of exactly 100 mL of drink. Manufacturer values for nutrient content per 100 mL were then used to determine water content per mL and per g, which were used to determine the weight of each beverage needed to provide exactly 1000 mL of water. This meant that the weight of each drink provided was slightly different, with each 250 mL water provided in 274.2 g (264.8 mL) MILK and 271.3 g (265.7 mL) SOYA. Given that urine output and fluid balance responses between conditions were the primary outcomes, this approach ensured the water consumed was identical between trials. Beverages were stored in a refrigerator (4–8 °C) until the start of the drinking period.

**Table 1:** Composition per litre of the MILK and SOYA beverages used in the study

	MILK	SOYA
Energy (kcal)	371	392
Protein (g)	36	30
Carbohydrate (g)	50	25
Fat (g)	3	18
Fibre (g)	0	5
Water (g)	944	941
Sodium (mmol)	17 (1)	9 (1)
Potassium (mmol)	45 (1)	65 (2)
Chloride (mmol)	27 (0)	7 (1)

Energy and macronutrient composition obtained from manufacturer information. Sodium and potassium concentrations for each beverage were analysed via flame photometry. Chloride concentrations for each beverage were analysed coulometric titration. Electrolyte data are mean (SD).

### Sample handling and analyses

For each urine sample, participants completely emptied their bladder into a clean, 1000 mL urine collection bottle. The weight of each urine sample was measured to the nearest 0.1 g (Kern PFB 2000, Thornaby, Stockton-on-Tees, UK), before urine specific gravity was measured (ATAGO, PAL-10S, Southam, Warwickshire, UK). An aliquot of each urine sample, and beverages from each trial, were retained at − 80 °C until analysis for sodium and potassium concentration by flame photometry (M410C Flame Photometer, Sherwood Scientific Ltd., Cambridge, UK; CV = 1.5 and 1.6%, respectively) and chloride concentration by coulometric titration (Sherwood Scientific 926S Chloride Meter, Sherwood Scientific Ltd., Cambridge, UK; CV = 0.5%).

### Statistical analyses

Statistical analyses were performed using IBM SPSS Statistic v27 and Microsoft Excel. Data and individual difference values were checked for normality using a Shapiro–Wilk test (with *P* < 0.05 considered not normally distributed). Data containing two factors (trial [2 levels] and time [5 levels]) were then analysed using a two-way repeated-measures ANOVA, whilst data containing one factor (trial) were analysed using paired *t* tests or Wilcoxon Signed-Rank tests, as appropriate (beverage palatability ratings). Where significant interaction effects were observed, differences between trials were explored using *post-hoc* paired *t* tests or Wilcoxon Signed-Rank tests, as appropriate. The familywise error rate was controlled using the Holm–Bonferroni correction. Data were normally distributed and presented as mean (standard deviation [SD]), apart from the subjective Likert scales, which were non-normally distributed and presented as median (interquartile range [IQR]). Statistical significance was set at *P* < 0.05.

## Results

### Pre-trial measurements and laboratory conditions

There were no differences between trials for pre-trial body mass (MILK, 78.75 (8.51) kg; SOYA, 78.51 (8.72) kg; *P* = 0.257), urine specific gravity (MILK, 1.008 (0.004); SOYA, 1.008 (0.004); *P* = 0.954), thirst (MILK, 5 (2); SOYA, 5 (2); *P* = 0.823) or any other subjective variable (*P* ≥ 0.317). There was no difference in average laboratory temperature (MILK, 22.7 (1.4) °C; SOYA, 23.1 (1.8) °C; *P* = 0.478) and relative humidity (MILK, 27.4 (8.1) %; SOYA, 27.1 (9.0) %; *P* = 0.940) between trials.

### Urine output and specific gravity

**Fig. 1:**
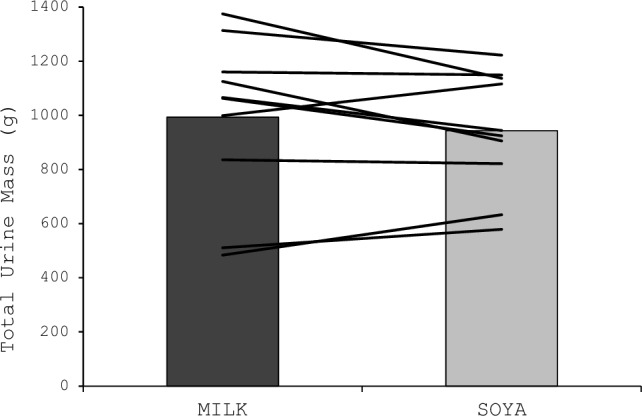
Total urine mass (g) over 210 min (30 min drinking and 180 min follow-up) following the consumption of MILK and SOYA beverages. Bars represent group mean; lines represent individual participants

**Fig. 2:**
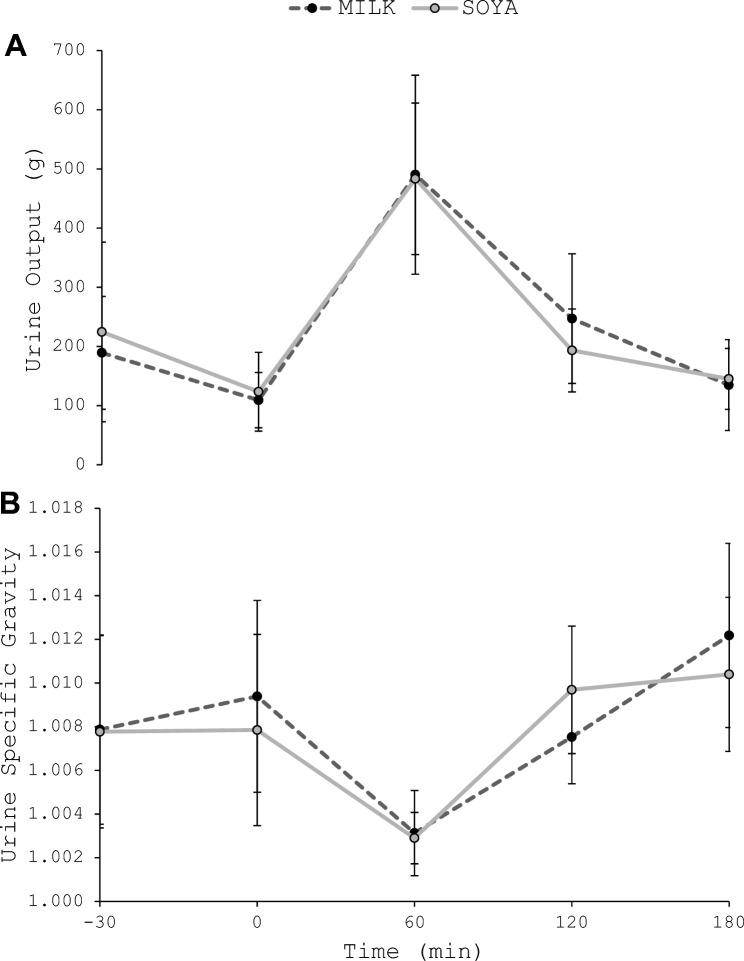
**A** Urine output (g) and **B** urine specific gravity throughout experimental trials. *Both trials different to − 30 min (*P* < 0.001)

Total urine mass over the 210 min (30 min drinking + 180 min follow-up) was not different between trials (*P* = 0.435; Fig. 1). There were no interaction effects for urine mass (Fig. 1a; *P* = 0.471) or urine specific gravity (Fig. 1b; *P* = 0.156), but there were main effects of time for both (*P* < 0.001). Compared to baseline, urine mass was greater (*P* < 0.001) and urine specific gravity lower (*P* < 0.001) at 60 min, with no other differences observed (*P* ≥ 0.373). There was no trial order effect for total urine mass (TRIAL 1, 993 (287) g; TRIAL 2, 943 (208) g; *P* = 0.270) (Fig. 2).

### Electrolyte excretion and balance

The MILK beverage was higher in sodium and chloride, but lower in potassium concentrations (all *P* < 0.001; Table 1). For cumulative urine electrolyte excretion (Table 2), there were no interaction effects for sodium (*P* = 0.174) or chloride (*P* = 0.257), but there was an interaction effect for potassium (*P* = 0.005), with greater potassium excretion in SOYA from 60 min onwards (*P* ≤ 0.048). When all three electrolytes were combined, there was no interaction effect for total electrolyte excretion (*P* = 0.631).

**Table 2:** Cumulative electrolyte excretion in urine after ingestion of 1,000 mL water from skimmed milk (MILK) and a sweetened soya beverage (SOYA)

Time (min)	− 30	0	60	120	180
Sodium (mmol)					
MILK	0	6 (3)	17 (8)	32 (13)	41 (16)
SOYA	0	4 (2)	13 (7)	24 (14)	30 (19)
Potassium (mmol)					
MILK	0	5 (2)	15 (5)	31 (9)	43 (12)
SOYA	0	6 (2)	19 (7)^#^	38 (10)^#^	55 (14)^#^
Chloride (mmol)					
MILK	0	7 (3)	24 (10)	45 (14)	60 (17)
SOYA	0	7 (3)	21 (8)	39 (14)	52 (18)
Total (mmol)					
MILK	0	18 (7)	56 (21)	107 (34)	144 (40)
SOYA	0	17 (6)	53 (18)	101 (28)	137 (36)

Data are presented as mean (SD)

^#^Indicates significant difference between MILK and SOYA (*P* < 0.05)

**Fig. 3:**
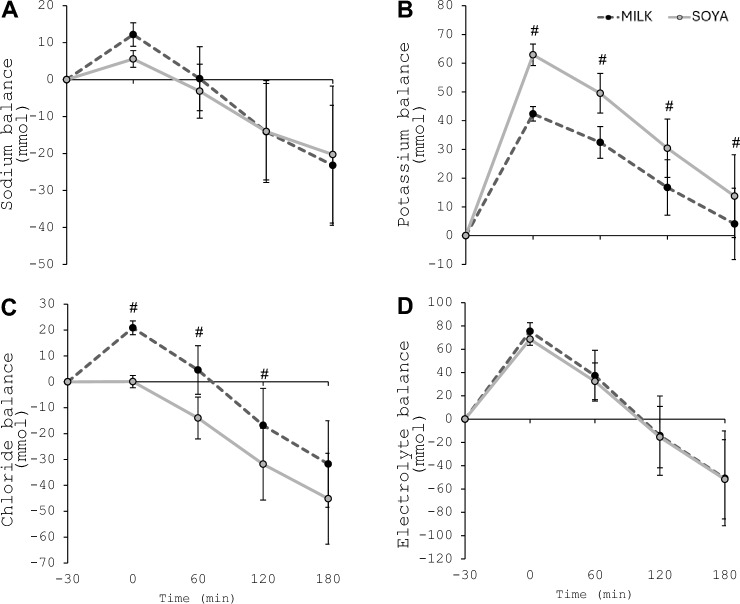
**A** Sodium; **B** potassium; **C** chloride, and **D** total electrolyte balance throughout experimental trials. ^#^Indicates significant difference between MILK and SOYA (*P* < 0.05)

There were interaction effects for potassium (*P* < 0.001; Fig. 3B) and chloride (*P* = 0.023; Fig. 3C) balance, but not sodium balance (*P* = 0.258; Fig. 3A). Potassium balance was greater in SOYA at all time points post-beverage consumption (*P* ≤ 0.013), whilst chloride balance was greater in MILK from 0 to 120 min (*P* ≤ 0.036), but not at 180 min (*P* = 0.068). When all three electrolytes were combined, there was no interaction effect for total electrolyte balance (*P* = 0.688; Fig. 3D).

### Subjective responses and beverage palatability

There were no interaction effects for subjective measures of hunger (*P* = 0.656), thirst (*P* = 0.139), nausea (*P* = 0.353), or stomach fullness (*P* = 0.448), although there were main effects of time for hunger, thirst, and stomach fullness (*P* < 0.001; Table 3).

**Table 3:** Subjective feelings of hunger, thirst, nausea and stomach fullness after ingestion of 1,000 mL water from skimmed milk (MILK) and a sweetened soya beverage (SOYA)

Time (min)	− 30	0	60	120	180
Subjective feelings (0–10 au)					
Hunger					
MILK	6 (4, 6)	5 (2, 6)	6 (3, 6)	7 (3, 7)	8 (5, 8)
SOYA	5 (4, 6)	5 (0, 5)	5 (4, 6)	6 (3, 7)	7 (5, 8)
Thirst					
MILK	5 (3, 7)	3 (0, 5)	5 (2, 6)	5 (3, 7)	6 (4, 8)
SOYA	4 (4, 7)	2 (0, 3)	5 (2, 6)	5 (3, 7)	7 (4, 8)
Nausea					
MILK	0 (0, 0)	1 (0, 2)	0 (0, 0)	0 (0, 0)	0 (0, 0)
SOYA	0 (0, 0)	0 (0, 2)	0 (0, 0)	0 (0, 1)	0 (0, 1)
Stomach fullness					
MILK	3 (1, 5)	8 (5, 8)	5 (3, 5)	3 (2, 4)	2 (1, 3)
SOYA	3 (2, 4)	7 (5, 8)	4 (3, 6)	3 (2, 5)	2 (1, 4)

Data are presented as median (quartile 1, 31)

*au* arbitrary unit, *IQR* interquartile range

There were no significant differences for how pleasant (MILK 6 (4, 7); SOYA 7 (3, 7); *P* = 0.866), thirst quenching (MILK 7 (6, 7); SOYA 6 (5, 7); *P* = 0.199) or refreshing (MILK 6 (5, 8); SOYA 4 (4, 7); *P* = 0.056) the beverages were rated.

## Discussion

In contrast to our hypothesis, this study found no significant difference in total urine output following consumption of skimmed milk or a sweetened soya beverage. There were also no differences in the pattern of urine output or urine concentration between the beverages. These results suggest that for those who do not consume cow’s milk in their diet, consumption of a sweetened soya beverages should not negatively impact their fluid balance, at least in healthy young males.

Maughan et al. (2016) compared fluid balance responses to 13 commonly consumed beverages, reporting that cow’s milk (skimmed and whole), and a specifically designed oral rehydration solution, were the only beverages that resulted in greater fluid balance compared to still bottled water (measured by the beverage hydration index [BHI]). In contrast, the BHI of the other beverages, including sugar-sweetened beverages, diet soft drinks, orange juice, coffee, tea, beer etc. were not different from water, once the difference in water content of the beverages was accounted for Maughan et al. (2016). BHI is defined as the urine output in the 2 h after ingesting 1000 mL of a beverage relative to the urine output in the 2 h after ingesting 1000 mL of still water (Maughan et al. 2016). Whilst it is not possible to calculate the BHI of the soya beverage due to the absence of a water trial in the present study, the finding that urine output was not different between skimmed cow’s milk and a soya beverage means the two beverages would elicit comparable BHIs. Indeed, at 2 h (i.e., when BHI is typically calculated), mean urine output was lower, albeit not significantly, in the soya trial [i.e., 850 (221) g vs 804 (213) g; *P* = 0.200], and 7 of 10 participants produced less urine up to 2 h in the soya trial. These values for urine output are comparable to values reported for skimmed and whole milk by Maughan et al. (2016), again supporting the notion that the soya beverage would produce a comparable BHI to milk.

Whilst the macronutrient and electrolyte content of the two beverages was not identical, the sodium and protein contents were similar, which are the variables most likely to influence hydration outcomes in this context (Evans et al. 2017; James 2013). Electrolytes are vital for regulating fluid balance and fluid distribution between body water compartments (Knepper et al. 2015). Whilst increased sodium concentration increases beverage retention (Maughan and Leiper 1995; Merson et al. 2008; Shirreffs and Maughan 1998) at concentrations found in the beverages of the present study (i.e. < 20 mmol/L), sodium appears to have little effect on fluid balance (Maughan et al. 2016, 2019). Whilst there was a larger difference in potassium content, potassium seems to have an equivocal effect on fluid balance, with studies comparing beverages of 30–71 mmol/L with potassium-free beverages reporting comparable fluid balance responses (Perez-Idarraga and Aragon-Vargas 2014; Shirreffs et al. 2007).

Milk protein added to post-exercise rehydration beverages independently increases fluid balance (James et al. 2011), with 2% milk protein appearing to be sufficient to maximise this effect (James et al. 2013). Using the mean data for urine volume at the end of the study of James et al. (2011), the milk protein beverage would have a BHI (in a post-exercise setting) of ~ 1.3 relative to the carbohydrate-only beverage, suggesting the protein content of milk plays a major role in its beneficial BHI. Whilst no study, to date, has isolated the effect of soya protein, one study reported comparable post-exercise rehydration between a soya-based beverage and whole cow’s milk (Desbrow et al. 2014). The two major proteins in soya are conglycinin and glycinin (Žilić et al. 2011), with soya typically containing ~ 40% β-conglycinin, which has also been demonstrated to reduce gastric emptying rate in animal models (Nishi et al. 2003). Slower gastric emptying increases fluid balance (Clayton et al. 2014; Evans et al. 2011), so it is possible that the soya beverage might benefit fluid balance via the effects of soya protein on gastric emptying (Evans et al. 2017). Given the variability in protein content of common plant-based dairy-milk alternatives, it seems unlikely other plant-based dairy-milk alternatives, typically with a much lower protein content than soya beverages, would illicit similar responses, but this should be examined in future studies.

The carbohydrate concentrations of the beverages were low (i.e., 5% in MILK and 2.5% in SOYA), and might not be expected to influence fluid balance, given that Maughan et al. (2019) reported that only a 20% sucrose beverage increased BHI, and not 5% or 10% sucrose beverages (Maughan et al. 2019). However, it is possible the carbohydrate in cow’s milk (lactose) could explain some of its beneficial hydration effect. Berry et al. (2020), reported that a milk permeate beverage containing 4% carbohydrate (2% glucose, 2% galactose), but no protein or fat, reduced urine output and increased BHI compared to water and a 6% carbohydrate (sucrose/glucose) beverage. Thus, the lactose in the milk may enhance fluid balance after milk consumption (Maughan et al. 2016, 2019; Shirreffs et al. 2007), but this is unlikely the case for the soya beverage, which contained no lactose/galactose. Therefore, the finding that milk and soya beverages were not different for hydration outcomes, despite the beneficial effects of milk protein (James et al. 2011, 2013) and galactose (Berry et al. 2020), suggests the soya proteins may be providing a comparable positive effect. There was a difference in fat content of the beverages (3 vs 18 g/L), but it is unlikely that this would influence outcomes. For example, Maughan et al. (2016) compared skimmed and whole cow’s milk (1 vs 36 g/L fat) and reported no difference in fluid balance outcomes or BHI, despite a larger difference in fat intake than in the present study.

There were no differences for hunger, thirst, satiety, or palatability scores between beverages, suggesting that the sugar-sweetened soya beverage provides an effective alternative to cow’s milk with regards to subjective outcomes. Although not measured in the present study, these subjective responses suggest substituting skimmed milk with a soya beverage would be unlikely to influence voluntary intake, with potential important implications for total daily fluid intake.

Only healthy, young (19–39 y) male participants were included in the final sample, so whether the results can be generalised to females, children or older adults is unclear. One previous study (Sollanek et al. 2018), reported that biological sex did not influence BHI or fluid balance responses to water, amino acid-containing or glucose containing beverages consumed at rest. Additionally, the menstrual cycle does not appear to influence fluid balance responses after exercise (Rodriguez-Giustiniani and Galloway 2019). Therefore, there is no reason to suspect these results would not translate to females, but future studies should examine this. In contrast, Rodriguez-Sanchez and Galloway (2023) reported that whilst milk contributes to maintaining a positive fluid balance compared to water in young adults (~ 25 y), it does not in older adults (~ 64 y). Therefore, whether the results of the present study translate to older adults is unclear. This is particularly important as older adults are more prone to hypohydration (Allison and Lobo 2004), meaning if a soya beverage can increase fluid balance in older adults, it could make a meaningful difference to maintenance of body water, with potential effects on various health outcomes. However, future studies should examine this, as well as similar outcomes in children/adolescents.

## Conclusion

In summary, there was no difference in total urine output following consumption of 1000 mL of water from skimmed cow’s milk or a sweetened soya beverage, indicating the sweetened soya beverage was as effective at maintaining fluid balance as skimmed cow’s milk in healthy young males. These results suggest that, for those who do not consume cow’s milk in their diet, replacing cow's milk with a sweetened soya beverage should not negatively impact their fluid balance, provided total water intake is not affected. Future research should examine the mechanisms behind the hydration potential of soya-based beverages, as well as comparing the effects to those of other plant-based milk alternatives.

## Data Availability

Data supporting these findings is available from the corresponding author upon reasonable request.

## Abbreviations

BHI: Beverage hydration index
SD: Standard deviation
CV: Coefficient of variation
ANOVA: Analysis of variance
IQR: Interquartile range
